# Contribution of the Immune Response in the Ileum to the Development of Diarrhoea caused by Helminth Infection: Studies with the Sheep Model

**DOI:** 10.1007/s10142-022-00864-6

**Published:** 2022-05-16

**Authors:** Shamshad Ul Hassan, Eng Guan Chua, Parwinder Kaur, Erwin A. Paz, Chin Yen Tay, Johan C. Greeff, Shimin Liu, Graeme B. Martin

**Affiliations:** 1 UWA School of Agriculture and Environment, The University of Western Australia, Crawley, WA 6009 Australia; 2 Helicobacter Research Laboratory, The Marshall Centre for Infectious Disease Research and Training, School of Biomedical Sciences, University of Western Australia, Perth, WA Australia; 3 Department of Primary Industries and Regional Development, Western Australia, 3 Baron Hay Court, South Perth, WA 6151 Australia

**Keywords:** Ileum, helminths, RNA-Seq, inflammatory immune response, diarrhoea, Th2 immunity

## Abstract

Gastrointestinal helminths are a global health issue, for humans as well as domestic animals. Most studies focus on the tissues that are infected with the parasite, but here we studied the ileum, a tissue that is rarely infected by helminths. We tested whether inflammation in the ileum contributes to the development and severity of diarrhoea, by comparing sheep that are susceptible (n = 4) or resistant (n = 4) to the disease. We analyzed the ileum transcriptome using RNASeq sequencing approach and various bioinformatics tools including FastQC, STAR, featureCounts, DESeq2, DAVID, clusterProfiler, Cytoscape (ClusterONE) and EnrichR. We identified 243 differentially expressed genes (DEGs), of which 118 were up-regulated and 125 were down-regulated DEGs in the diarrhoea-susceptible animals compared to the diarrhoea-resistant animals. The resulting DEGs were functionally enriched for biological processes, pathways and gene set enrichment analysis. The up-regulated DEGs suggested that an inflammatory immune response was coupled with genes involved in ‘Th2 immune response’ and ‘anti-inflammatory response’. The down-regulated DEGs were related to ion transport, muscle contraction and pathways preventing inflammation. We conclude that i) susceptibility to helminth-induced diarrhoea involves an inflammatory response at a non-infectious site; ii) down-regulation of pathways preventing inflammation can contribute to the severity of diarrhoea; and iii) genes involved in anti-inflammatory responses can reduce the inflammation and diarrhoea.

**Supplementary Information:**

The online version contains supplementary material available at 10.1007/s10142-022-00864-6.

## Introduction

Gastrointestinal helminths are a global health issue, for humans as well as domestic animals, including sheep, where helminth-induced diarrhoea is a major cause of production losses (Taylor 2012). We have been studying helminth–sheep interactions in a Mediterranean region where winter rainfalls are dominant and are conducive to infection of Merino sheep by *Teladorsagia circumcincta* and *Trichostrongylus colubriformis*, the major causes of diarrhoea, or ‘winter scouring’ (Jacobson et al. 2020). The climate favours the development of L3 stage larvae on pastures from where they are ingested by sheep as they graze. In the gastrointestinal tract (GIT), the larvae damage the mucosae, develop to adult stage and start laying eggs that are then passed in the faeces and deposited on the pasture where they hatch, thus completing the life cycle (Karlsson et al. 2004).

After ingestion, *T. circumcincta* primarily infects the abomasum (the equivalent of the stomach of non-ruminant animals), whereas *T. colubriformis* infects the duodenum (the first few metres of the small intestine; (Craig 2009) predilection sites (sites preferred by helminths) have generally been the focus for studies of the disease process using histopathology, immunohistochemistry and gene expression analysis. Consequently, very little is known about the roles of other parts of the GIT, such as the ileum (the final segment of the small intestine), in the development, persistence and severity of helminth-induced diarrhoea.

The processes leading to diarrhoea are multifactorial and include the damage to the GIT epithelium by the invading helminths, disruption of ion transport and fluid absorption of due to changes in gut motility and permeability (Williams et al. 2010; Williams and Palmer 2012) and, in some cases, an ‘inflammatory immune response’ (Jacobson et al. 2020; Karlsson et al. 2005; Larsen et al. 1994). The immune response to GIT helminths involves both the innate and the adaptive components of immune system, and they combine to expel and resist infection. Ingestion of L3 stage larvae first provokes the innate immune component, leading to the presentation of physical barriers (increases in smooth muscle contraction; thickening of the mucous layer and production of defensins; (Douch et al. 1984; Harrison et al. 1999; Harrison et al. 2003), the recruitment of cytotoxic and pro-inflammatory cells (mast cells, eosinophils; (Henderson and Stear 2006; Balic et al. 2006; Buddle et al. 1992), activation of pattern-recognition receptors (e.g. toll-like receptors, C-type lectin receptors expressed by antigen-presenting immune cells; (Ingham et al. 2008) and production of chemoattractant molecules (the eotaxin family of chemokines, including CCL11, CCL24 and IL-5; (Rosenberg et al. 2013). The innate immune response leads to activation of the more precise adaptive immune response by presenting helminth antigens to specialized molecules, from the major histocompatibility complex (MHC-II), that are present on antigen-presenting cells. This outcome leads to infiltration of mast cells and eosinophils, activation of the Th2-mediated (T-helper cell type 2) antibody response (IgA/IgE), through the agency of Th2 cytokines (IL-4, IL-5, IL-13), ultimately resulting in reduced establishment of larvae, helminth expulsion or reduced helminth fecundity (McRae et al. 2015). If the helminth burden overcomes the immune response, diarrhoea can result. Alternatively, the host can become susceptible to helminth infection if a Th1-mediated (T helper cell type 1) immune response is initiated with involvement of Th1 cytokines (INF-γ, IL-12) and CD8^+^ cytotoxic T cells (Finkelman et al. 1994; Gill et al. 2000; Craig et al. 2014).

In our previous work, we investigated the haematology profiles of sheep that were genetically susceptible or resistant to the development of diarrhoea (assessed by diarrhoea score and faecal consistency score) and of sheep that were genetically resistant or susceptible to helminth infection (assessed by faecal worm egg count). The haematology profiles included haemoglobin content, packed cell volume, red blood cell count and white blood cell count. The results led us to conclude that haematological profile does not explain susceptibility or resistance to infection or diarrhoea, and thus offer no promise as traits for genetic selection (Greeff et al. 2020). We then investigated the transcriptomes of the duodenum (the site of infection) where we compared the expression profiles of genes involved in the immune responses, again comparing diarrhoea-susceptible diarrhoea-resistant animals to test whether a hypersensitive inflammatory or immune response explains diarrhoea in animals with a low worm burden (Hassan et al. 2022). Our observations made it clear that the mechanisms of immune suppression, tissue repair and maintenance of physical barriers were activated in diarrhoea-susceptible sheep, leading us to reject the hypersensitivity hypothesis. Conversely, a Th2 immune response was activated in diarrhoea-resistant sheep, explaining their phenotype.

These studies led us to question whether another section of the intestine, where infection does not occur, would present support for an inflammatory/hypersensitive immune response. The ileum came into focus because our histological analysis showed that this intestinal segment had more eosinophils and masts cells than to the other sections of the GIT (Niu et al. 2021). Therefore, in the present study, we tested whether the expression of the genes that control the inflammatory immune response is related to the development and severity of diarrhoea after helminth infection in sheep.

## Materials and Methods

### Animal source, experimental design and Ethics statement

The lambs used in this experiment were sourced from the flocks that had been maintained at the Katanning Research Facility in Western Australia since 2015 and, prior to that, at the Mount Barker Research Station, as part of a long-term breeding experiment addressing the helminth-diarrhoea-flystrike complex (Greeff et al. 2020). The Katanning Research facility is situated at an altitude of 300 m, latitude of 33.7°S and longitude 117.55°E, and experiences 480 mm yearly rainfall, with a winter distribution characteristic of a Mediterranean climate. This study was sanctioned by the Animal Ethics Committee of the Department of Primary Industries and Regional Development, Western Australia (AEC No.17-1-02 v 2.1).

In November 2016, 986 lambs at weaning age (about 3 months) were sampled for faecal worm egg count (FEC), administered a broad spectrum anthelmintic (Monopantel® @ 1 mL/10 Kg body weight), scored for diarrhoea (‘dag score’, an indicator of the amount of faecal material accumulated on the hind quarters between hock joint and anus; scale of 1–5 with 5 being a high). These data and similar data from previous generations were submitted to Sheep Genetics (www.sheepgenetics.org.au) to obtain estimates of the Australian Sheep Breeding Values (ASBV) for ‘dag score’ and FEC. The ASBV values for ‘dag score’ were used to identify 100 males and 100 females that were most or least susceptible to diarrhoea.

Males and females managed similarly but were kept in separate paddocks at a stocking rate of 10 animals/1000m^2^; here, they were allowed to graze winter-spring pasture composed primarily of *Trifolium subterraneum*, *Trifolium michelianum*, *Trifolium glomeratum* and *Trifolium repens*. To minimize contamination with helminth eggs, the paddocks were not grazed for 4 months before the start of experiment. Green feed would normally not become available until May, but in 2017 the sheep had access to green pastures from February to September due to unusual rainfall at the start of experiment. In this environment, the cycle of helminth infection is typically related to the onset of winter rains in April-May.

For all 200 lambs, diarrhoea and FEC were scored in March, May, June (males only), July (females only), August, and September, when the experiment ended. At the end of September, 20 diarrhoea-susceptible and 18 diarrhoea-resistant sheep were identified and slaughtered. Tissues from the GIT were sampled from four diarrhoea-susceptible and 4 diarrhoea-resistant sheep for study of gene expression. Helminth infection was verified by monitoring FEC monthly and at slaughter; moreover, at slaughter, helminths were counted and the dominant species were *T. circumcincta* in the abomasum and *T. colubriformis* in the small intestine, as reported in previously (Greeff et al. 2020). Helminths were generally absent from the ileum. These observations agree with previous studies showing that the predilection sites are the abomasum for *T. circumcincta (*McNeilly et al. 2009) and the first few meters of the duodenum for *T. colubriformis *(Wagland et al. 1996). We also monitored the health of the sheep, during the experiment and at slaughter, for symptoms related to causes of diarrhoea other than helminth infection. By monitoring FEC on monthly basis and counting helminth species in the GIT at slaughter, we excluded other causes of diarrhoea (protozoal, viral or bacterial). The tissue samples were stored in RNAlater (Sigma-Aldrich, St. Louis Missouri, USA) at –80°C, as per manufacturer’s instructions, until RNA extraction.

### RNA extraction, library preparation, quality control and sequencing

Total RNA was extracted from approximately 30 mg ileum tissue from each sheep using the RNeasy mini plus kit (Qiagen, Hilden, Germany), according to manufacturer’s instructions with minor modifications. Before extraction, *RNaseZAP* (Thermo Scientific™; Waltham, Massachusetts, USA) was used to decontaminate RNases on working surfaced and pipettors. The tissue samples were placed in 700 μL lysis buffer and homogenized using tissue Lyser-II (Qiagen, Hilden, Germany). The homogenate was centrifuged at 14,000 g for 3 min and the resulting supernatant was loaded onto genomic DNA (gDNA) removal columns and centrifuged at 10,000 g for 1 min. An equal volume of 70% (v/v) molecular grade ethanol was added to the flow-through from each gDNA column, mixed thoroughly by repeated pipetting, and loaded onto RNA binding columns, centrifuged at 10,000 g for 15 s. The flow-through was discarded and the columns were washed with 350 μL RW1 wash buffer. To ensure complete removal of gDNA, an additional step of on-column gDNA digestion was included, using DNase-I (0.34 Kunitz/μL; Qiagen, Hilden, Germany) for 15 min. The columns were washed again with 350 μL RW1 wash buffer before being washed twice with 500 μL of RPE buffer, followed by elution for the final time with 50 μL RNAse-free water.

A Qubit fluorometer (Thermo Scientific™; Waltham, Massachusetts, USA) was used to quantify RNA with an RNA-BR kit. The quality was checked on a 2% agarose gel and purity was checked using a NanoDrop 2000 (Thermo Scientific™; Waltham, Massachusetts, USA). The samples with (28S/18S) rRNA ratio greater than 1.5, an OD (260/280) ratio greater than 2 and an OD (260/230) ratio of 1.8 or greater were retained for further processing. The samples were sent to BGI-Hong Kong for library preparation and sequencing. To establish the RNA integrity number (RIN), samples were also analyzed on an Agilent Bioanalyzer-2100 (Agilent, Santa Clara, California, USA). Samples with RIN values ≥7 were further processed for library preparation and sequencing. All the libraries were sequenced with a depth of ≥ 22 million reads/sample using the pair-end approach (read length 100 base pairs) on a DNBseq^TM^ platform (BGI, Hong Kong).

### Bioinformatics analysis; identification of differentially expressed genes (DEGs)

The quality of the reads was checked by FastQC and reads with a score greater than 30 were aligned with the reference genome (Oar_rambouillet_v1.0) using STAR (v2.7.3a) (Dobin et al. 2013), a very robust and accurate alignment tool. Before alignment, indexes were created using the reference annotation gtf file (NCBI *Ovis aries* Annotation Release 103) and a reference genome (Oar_rambouillet_v1.0) fasta file. The featureCounts function in Subread software v2.0.0 (Liao et al. 2014) was used to count raw genes after mapping with the following parameters: *-t gene --primary -p.* The DESeq2 R package was used to study differentially expressed genes, using the diarrhoea-resistant group as the control (Love et al. 2014). A gene with a false discovery rate (FDR) cut-off < 0.05 and a log2fold change ≥ 1 was considered to be differentially expressed. A principal component analysis (PCA) was performed on the DESeq2-normalised count data after regularised log transformation to estimate and visualize the variations between samples.

### Functional enrichment of DEGs

A web-based tool, DAVID (The Database for Annotation, Visualization and Integrated Discovery) (Huang et al. 2009), was used to provide further information about the gene ontology (GO) terms that were significantly enriched in the DEGs. Additionally, the clusterProfiler (v3.18.1) R package was used for Gene Set Enrichment Analysis (GSEA) with org.Bt.eg.db (*Bos taurus*) being the source of annotation as the genome-wide annotation list for sheep was not available. The focus in GO terms was biological processes and KEGG pathways with a standard false discovery rate of less than 5% (FDR < 0.05). The Search Tool for the Retrieval of Interacting Genes/Proteins (STRING, v11) (Szklarczyk et al. 2019), a biological database and a web resource, was used to highlight functional interactions among the DEGs. This biological database collects and integrates all functional interactions between the proteins/genes by linking projected and known protein–protein interaction (PPI) data for many organisms. STRING was used to generate PPI with these options selected: interactions discarded with a confidence score < 0.4; ‘disconnected nodes hidden’ and ‘no more than 5 interactors to show in 1^st^ and 2^nd^ shell’. To cluster and visualize gene sub-networks in Cytoscape (v 3.7.2), the ClusterONE plugin was used that clusters genes by functional relevance. Clusters were used that had a P-value < 0.05 cut-off and a minimum > 5 genes per cluster. Another web-based tool, Enrichr, was used to study the pathways and biological functions related to genes in the sub-networks (Chen et al. 2013).

## Results

### RNA-Seq Data description and Principal component analysis

**Fig. 1:**
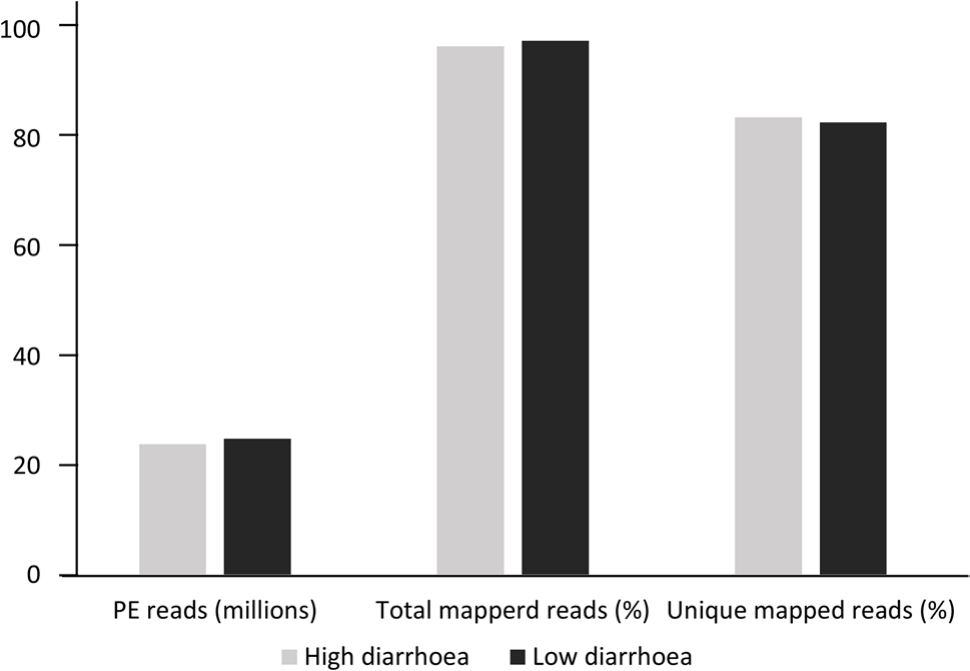
The mapping statistics from the RNASeq analysis of the ileum from sheep with high and low susceptibility to diarrhoea

The sequencing resulted in 198,340,385 pair-end reads with a range from 22,267,551 to 26,038,920 pair-end reads per sample and a minimum of 21,683,292 reads in each sample successfully mapped to the reference genome. Average unique mapping rates were 84% in the diarrhoea-susceptible group and 83% in the diarrhoea-resistant group (summarized in Fig. 1).

**Fig. 2:**
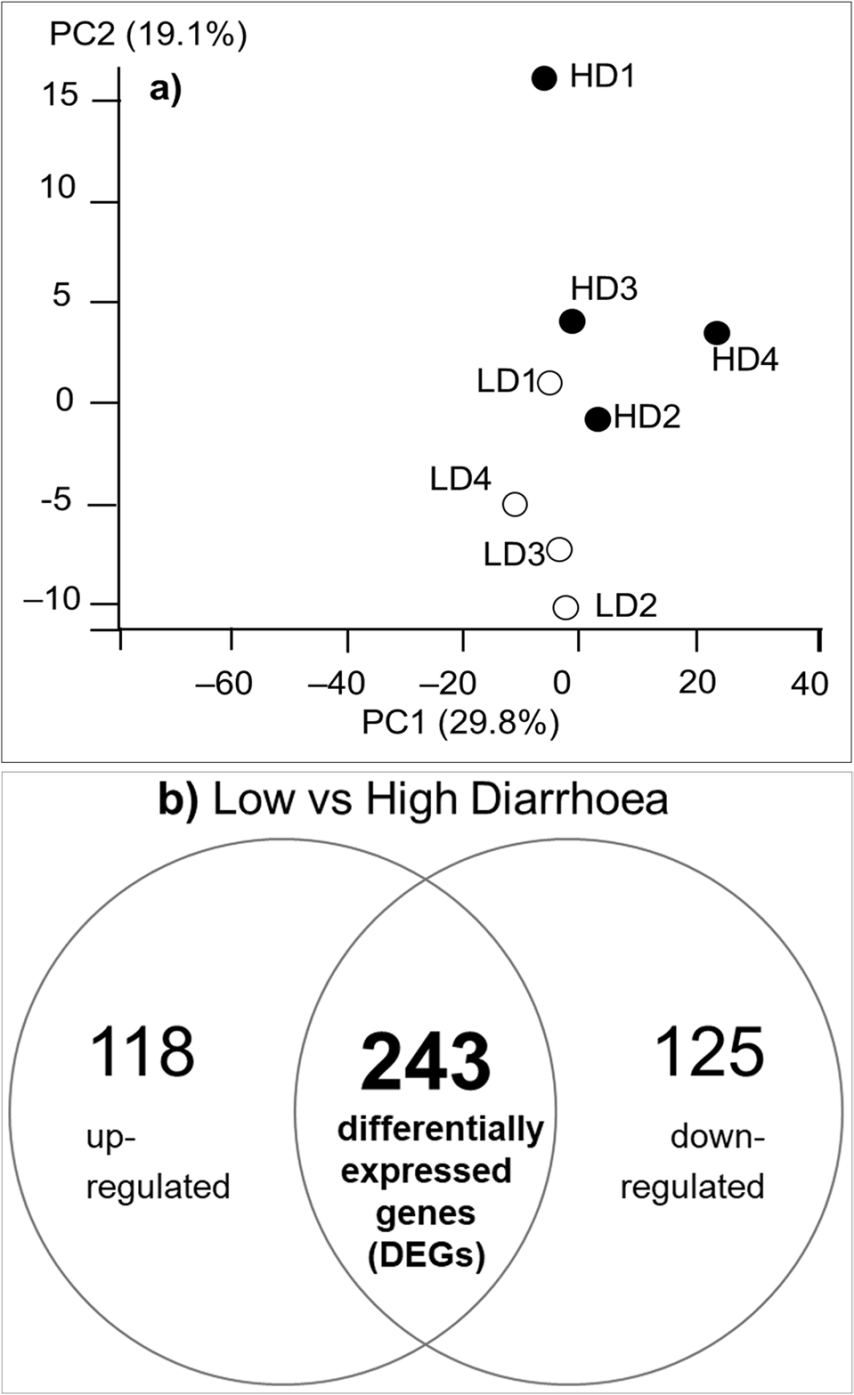
**(a)** Principal component analysis scatter plot showing gene expression in the ileum of the diarrhoea-susceptible (HD) and diarrhoea-resistant (LD) groups. The percentages on each axis represent the proportion of variation explained by the principal components, with PC1 (29.8%) being variation across groups and PC2 (19.1%) being variation within group. **(b)** Venn diagram showing the numbers of up-regulated and down-regulated genes in the diarrhoea-susceptible group, in comparison with the diarrhoea-resistant group. The total differentially expressed genes (DEGs) are indicated by the over-lapping area

The PCA plot (Fig. 2a) showed a tight clustering of the diarrhoea-susceptible samples, and a variable distribution of diarrhoea-resistant samples, with PC1 and PC2 representing 29.8% and 19.1% of the total variance, respectively, indicating inherent differences between diarrhoea-susceptible and diarrhoea-resistant sheep in gene expression in the ileum that can be explained through genetic variation.

### Comparative analysis of DEGs

**Fig. 3:**
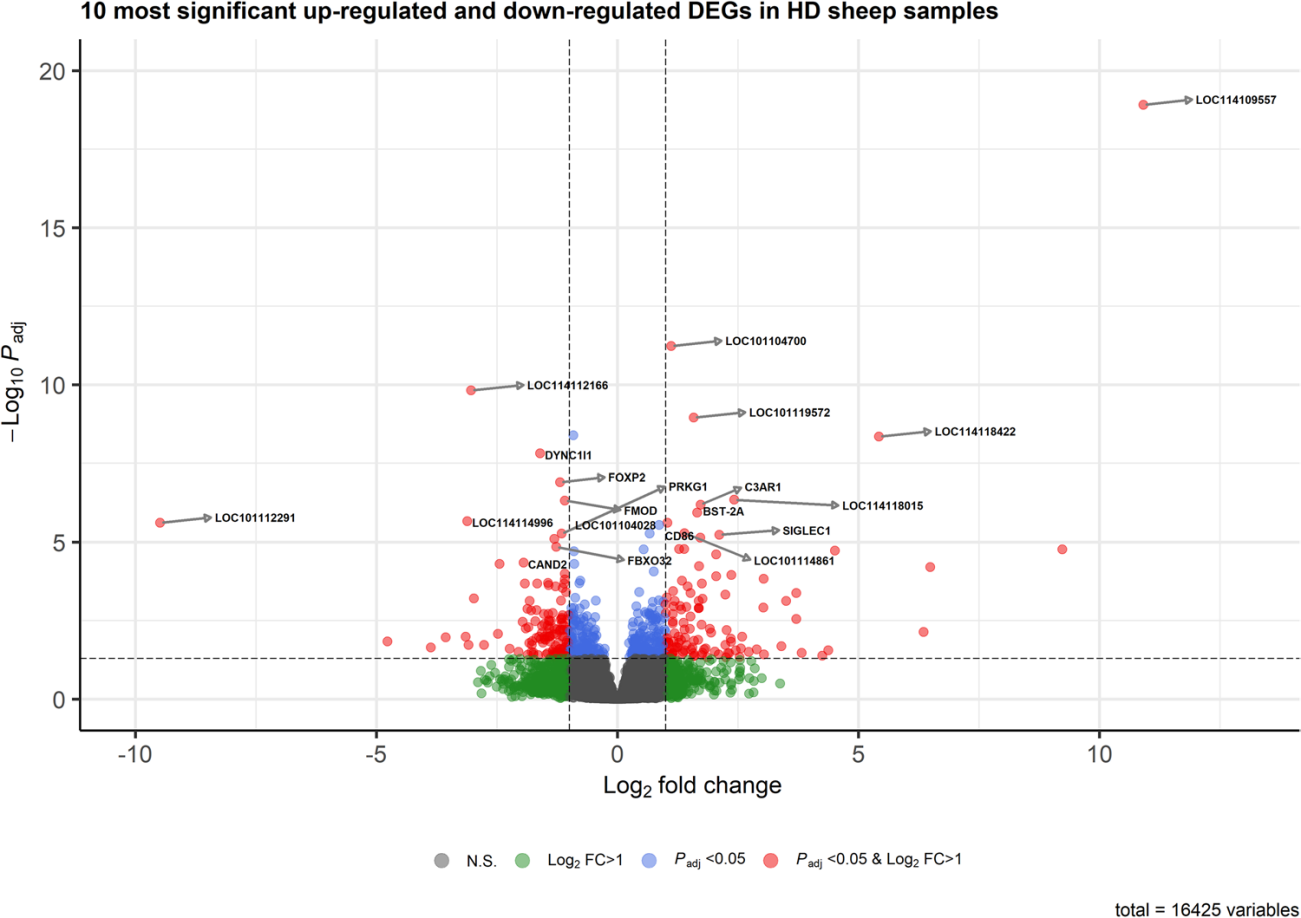
Volcano plot showing the 10 most significant DEGs that were up-regulated or down-regulated in the ileum of diarrhoea-susceptible sheep (HD) compared to diarrhoea-resistant (LD) sheep

We identified 243 DEGs, of which 125 were down-regulated and 118 were up-regulated in the diarrhoea-susceptible group in comparison with the diarrhoea-resistant (control) group (Fig. 2b). The details of all DEGs and associated biological processes and pathways are presented in Supplementary File S1, and a heatmap plot showing the top 100 DEGs is presented in Supplementary Fig. S1. The 10 most significant up-regulated and down-regulated DEGs are shown in Fig. 3. Among the up-regulated DEGs were CD86, SIGLEC1, C3AR1, BST-2A and several genes with functions that are not determined; the down-regulated DEGs included PRKG, FOXP2, FBX032, FMOD, CAND2, as well as a few genes with functions not yet determined.

### Functional enrichment, PPI network and sub-network analysis of DEGs

**Fig. 4:**
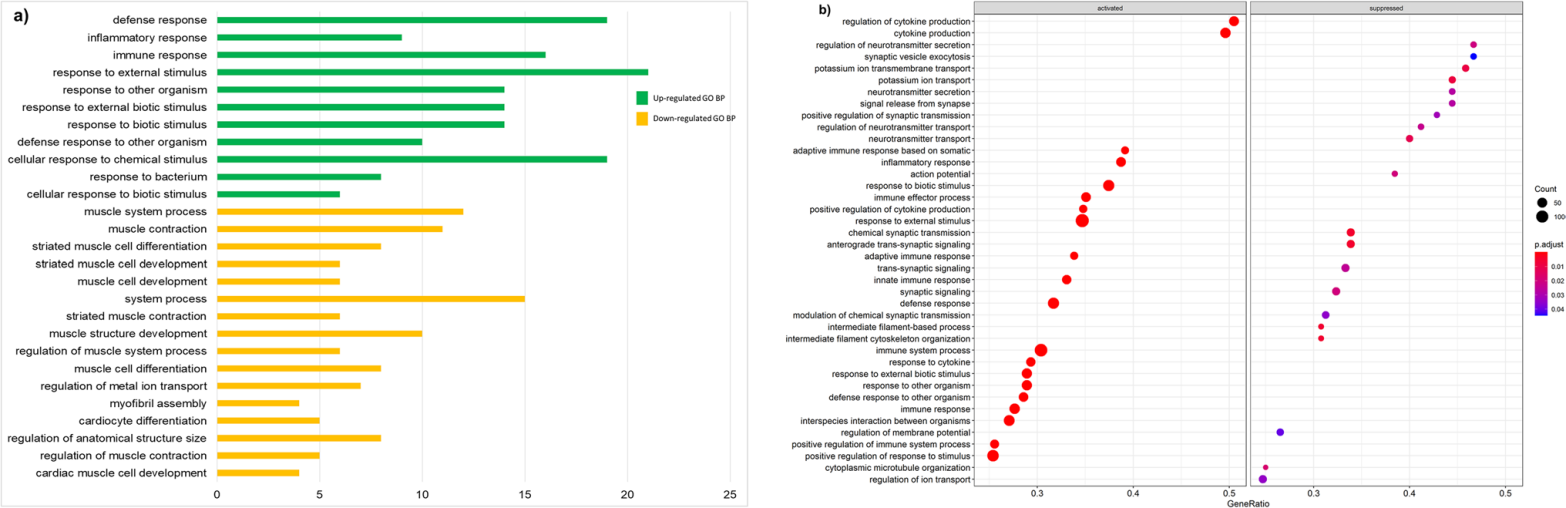
Functional enrichment analysis for GO biological process terms in the ileum of diarrhoea-susceptible sheep. **(a)** Functional enrichment analysis with DAVID using the up-regulated and down-regulated DEGs as the input data. The values on the x-axis represent the number of genes associated with each significant GO biological process term. **(b)** Functional enrichment analysis using GSEA of the entire DESeq2-normalised dataset. The top 40 significantly enriched GO biological process terms are shown

Functional enrichment of DEGs was based on GO biological processes and KEGG pathways, with FDR < 0.05 considered as significant using DAVID and GSEA in clusterProfiler (importantly, the outcomes were similar for the two analyses). In the diarrhoea-susceptible group, the common significant GO terms in the biological processes in the ileum included: ‘defense response’, ‘immune response’, ‘response to biotic stimulus’ and ‘inflammatory response’ (Fig. 4). The common significant KEGG pathways included ‘phagosome’, ‘tuberculosis’, ‘graft-versus-host disease’, ‘staphylococcus aureus infection’, ‘allograft rejection’, ‘type I diabetes mellitus’, ‘autoimmune thyroid disease’, ‘cell adhesion molecules (CAMs)’ and ‘rheumatoid arthritis’ (Supplementary File S1).

**Fig. 5:**
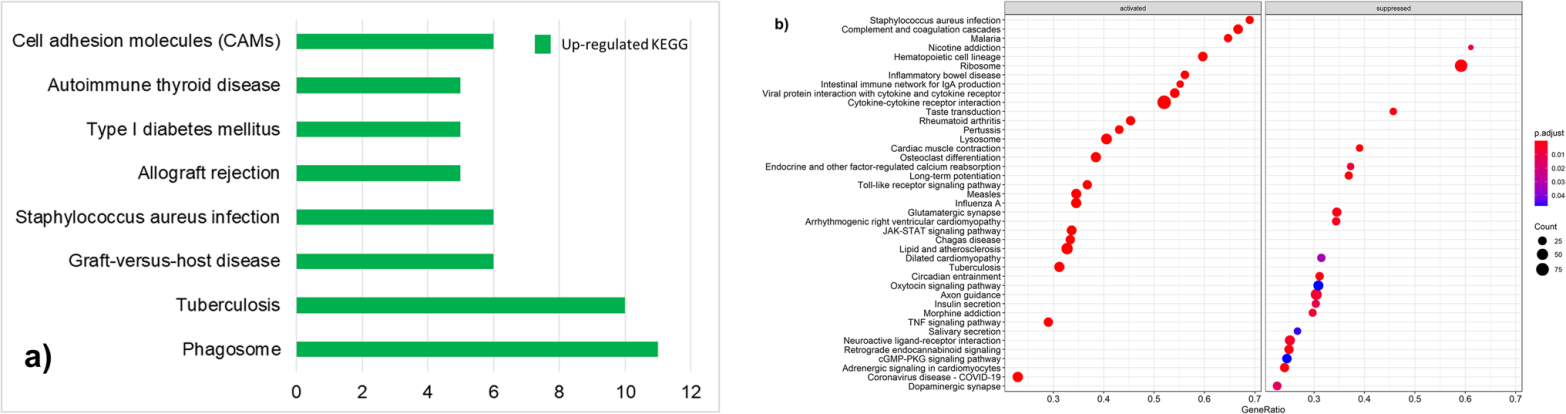
Functional enrichment analysis for KEGG pathways in the ileum of diarrhoea-susceptible sheep. **(a)** DAVID analysis using up-regulated and down-regulated DEGs as the input data. The values on the x-axis represent the number of genes associated with each significant KEGG pathway. **(b)** GSEA analysis using the entire DESeq2-normalised dataset. The top 36 significantly enriched KEGG pathways are shown

Importantly, GSEA analysis revealed significant up-regulation of the pathways for ‘inflammatory bowel disease’ and ‘complement and coagulation cascades’ in diarrhoea-susceptible sheep (Fig. 5b).

**Fig. 6:**
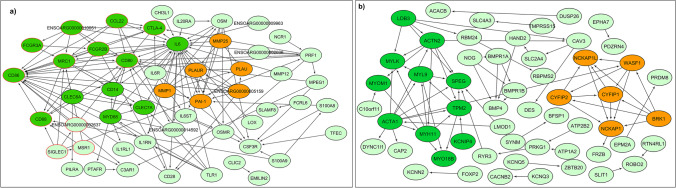
Protein–protein interaction (PPI) networks derived from STRING with sub-networks (SN) derived using the Cytoscape ClusterONE plugin. **(a)** Up-regulated DEGs: SN1 (dark green nodes); SN2 (red bordered nodes); SN3 (orange nodes). **(b)** down-regulated DEGs: SN1 (dark green nodes); SN2 (orange nodes)

The network analysis of the biological processes in the ileum in diarrhoea-susceptible sheep revealed three significant sub-networks of genes. Some of the enriched terms included ‘cellular response to molecule of bacterial origin’, ‘cellular response to lipopolysaccharide’, ‘positive regulation of type 2 immune response ’, ‘regulation of interleukin-6 production’, ‘inflammatory response’ and ‘positive regulation of inflammatory response’. It is clear from the functional enrichment of these sub-networks that they are associated with inflammation or bacterial infection (Supplementary File S2). The network and sub-networks for up-regulated genes are shown in Fig. 6a.

The significant GO biological processes associated with down-regulated DEGs in both analyses were ‘muscle system process’, ‘muscle contraction’, ‘striated muscle cell development and differentiation’, ‘muscle cell and structure development’, ‘regulation of metal ion transport’, ‘myofibril assembly cardiocyte differentiation’, ‘potassium ion transport’, ‘neurotransmitter transport’ and ‘regulation of ion transport’ (Fig. 4). The DAVID analysis revealed no significant KEGG pathways enriched in down-regulated DEGs (Supplementary File S1). The GSEA analysis revealed ‘cardiac muscle contraction, ‘cAMP signaling pathway’, ‘oxytocin signaling pathway’, ‘insulin secretion’, ‘salivary secretion’ and ‘endocrine and other factor-regulated calcium reabsorption’ as down-regulated pathways, among others (Fig. 5). The network analysis resulted in two significant sub-network of genes, and their functional enrichment was associated with ‘smooth muscle contraction’, ‘muscle fiber development’, ‘vascular smooth muscle contraction’ and ‘oxytocin signaling pathway’ (Supplementary File S2). The network and sub-networks for down-regulated genes are shown in Fig. 6b.

## Discussion

We were able to identify remarkable differences in the molecular mechanisms (pathways and biological processes) in the ileum of sheep that were associated with susceptibility to develop diarrhoea, in spite of the fact that the parasites of interest establish in the abomasum and duodenum, and not in the ileum. The diarrhoea-susceptible sheep show a dominant ‘inflammatory’ immune response with a Th2 polarity, whereas the diarrhoea-resistant sheep appear to express physiological states that promote smooth muscle contraction, while maintaining intestinal transport and absorption at homeostatic level.

**Fig. 7:**
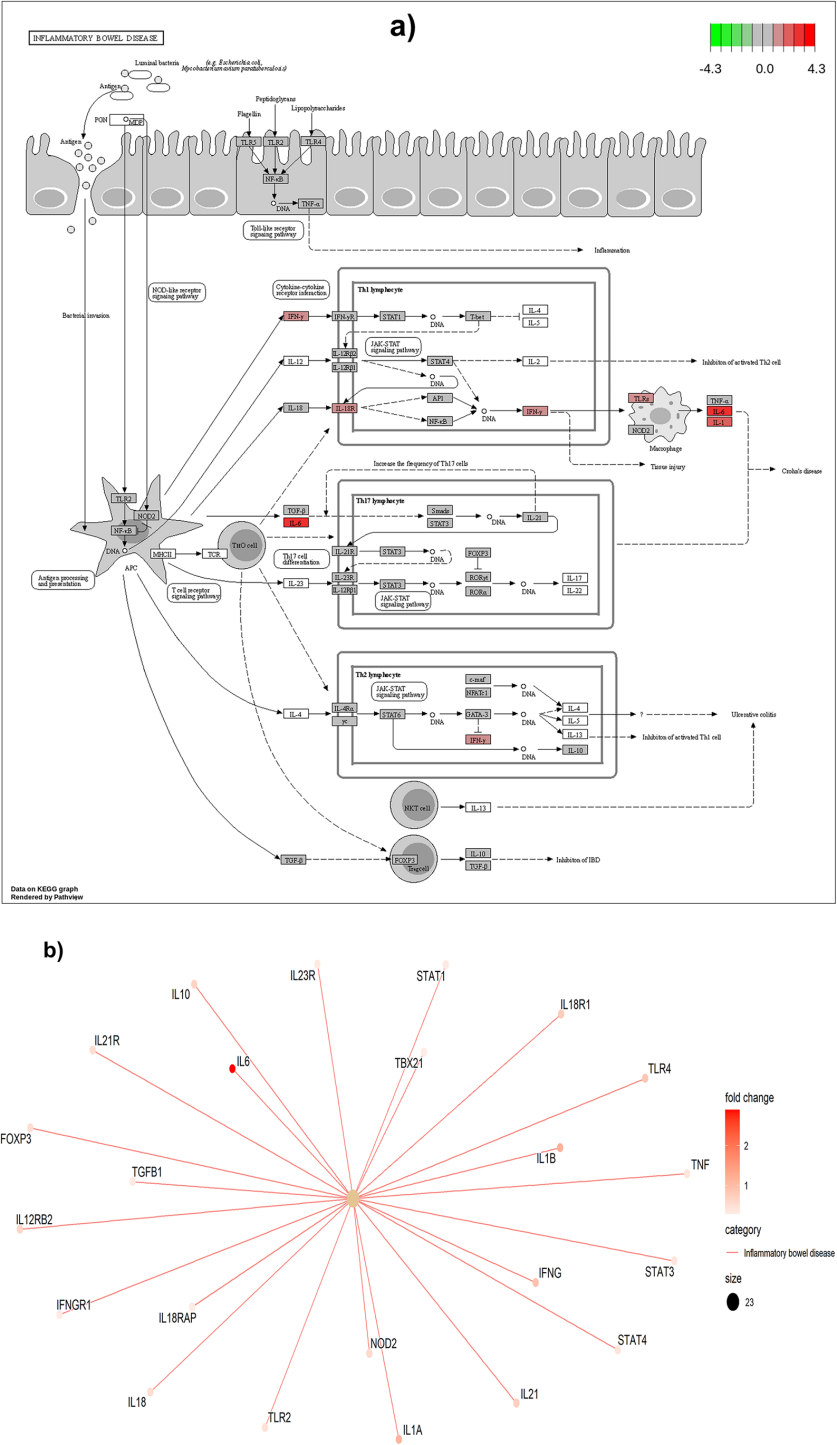
‘Inflammatory bowel disease’ KEGG pathway (Kanehisa et al. 2020) **(a)** with up-regulated genes in our study highlighted in red colour in the diarrhoea-susceptible group. **(b)** A category Netplot (CNET) showing the relationships among genes in the inflammatory bowel disease pathway

**Fig. 8:**
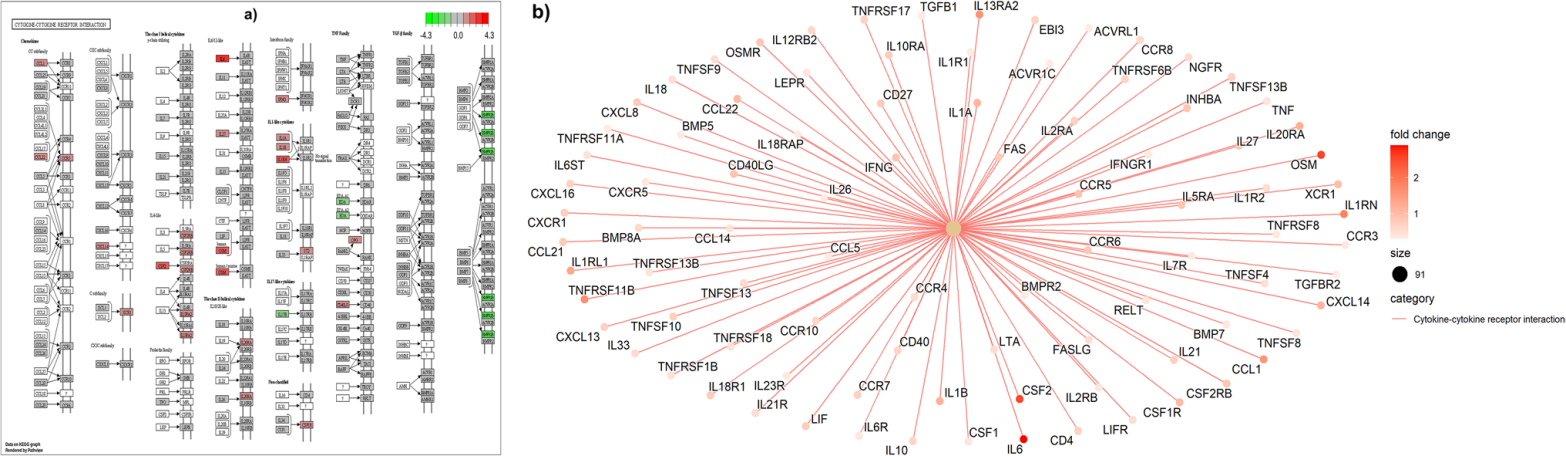
‘Cytokine–cytokine receptor interaction’ KEGG pathway (Kanehisa et al. 2020) **(a)** with up-regulated genes in our study highlighted in red and down-regulated genes highlighted in green colour in the diarrhoea-susceptible group. **(b)** A category Netplot (CNET) showing the relationships among the genes in cytokine–cytokine receptor interaction pathway

In the diarrhoea-susceptible sheep, the up-regulated genes were linked to the immune responses and inflammation, with the most important processes and pathways including ‘defense response’, ‘inflammatory response’, ‘inflammatory bowel disease’, ‘cytokine-cytokine receptor interaction’, ‘response to other organism’, ‘complement and coagulation cascades’, ‘phagosome’, ‘graft versus host disease’, ‘allograft rejection’, ‘autoimmune thyroid disease’, ‘rheumatoid arthritis’ and ‘cell adhesion molecules’. An interesting comparison can be made with human inflammatory bowel disease (IBD), such as Crohn’s disease, a complex disease with genetic disposition that involves an aberrant immune response towards intestinal pathogens and the microbiome (Graham and Xavier 2020), as shown in Fig. 7a, b. In the sheep ileum, diarrhoea susceptibility is linked to the ‘inflammatory response’ biological process and to the ‘inflammatory bowel disease’ KEGG pathway, similar to IBD in humans where diarrhoea is also linked to inflammation of the ileum (Larsen et al. 1999). Most studies of IBD are conducted on humans where risk factors have been identified, including nucleotide-binding oligomerization domain2 (NOD2), a gene revealed by GSEA analysis as being enriched in diarrhoea-susceptible sheep (Al Nabhani et al. 2017). NOD2 is an intracellular pattern recognition receptor and has been associated with an autoinflammatory disease marked by abnormally increased inflammation, predominantly mediated by cellular components of the innate immune system (Kastner et al. 2010; Yao et al. 2015). Cytokine–cytokine receptor interaction, another pathway up-regulated in diarrhoea-susceptible sheep, indicates interactions among cytokines (Elias and Zitnik 2012), particularly those that have been up-regulated in our study (Fig. 8a, b).

Some of the DEGs in diarrhoea-susceptible sheep, enriched in GO terms and sub-network analysis, included IL-6, LOC101121216 (serum amyloid A), SIGLEC1, CHI3L1, S100A9, CD14, CD68, CD86, Ovar-DRB1, IL1RL1, LOC101103238 (CXCL5), CCL22 and IL1RN. As helminth infection occurs at the abomasum and duodenum in our model, the up-regulation of immune system genes in the ileum was striking. IL-6, an important cytokine generally associated with inflammation and autoimmunity (Tanaka et al. 2014; Gabay 2006), can be both pro- and anti-inflammatory: *trans-signaling* (via a soluble form of IL-6 receptor with a broader spectrum of IL-6 target cells) leads to pro-inflammatory responses, whereas *classic signaling* (via membrane-bound IL-6 receptor) promotes anti-inflammatory and regenerative responses (Scheller et al. 2011). In our study, IL-6 has been enriched in inflammation-related biological processes and pathways, so we need to consider its inflammatory role at the ileum. Anti-IL-6 receptor antibody can be used treat chronic inflammatory conditions, such as rheumatoid arthritis (Tanaka et al. 2014; Scheller et al. 2011). IL-6 production can also be promoted by another gene that is up-regulated in diarrhoea-susceptible animals, serum amyloid A (SAA) another indicator of inflammation, a molecule with cytokine-like properties and immunomodulatory roles (Koga et al. 2008). It is clear that SAA has chemoattractant properties for T-lymphocytes, monocytes and leukocytes, and induces the secretion of pro-inflammatory cytokines, including IL-6, IL-8, TNF-α and IL-1β (Thompson et al. 2016; Badolato et al. 1994; Ebert et al. 2015). Other important genes with inflammatory outcomes, some of which were among top significantly up-regulated genes, were SIGLEC1 (Asano et al. 2015), C3AR1 (Banda et al. 2012; Coulthard and Woodruff 2015), S100A9 (Frohberger et al. 2020; Wang et al. 2018), CHI3L1 (Lee et al. 2009; Zhao et al. 2020), CXCL5 (Wang et al. 2009; Z’Graggen et al, 1997), CD14 (Grimm et al. 1995), CD68 (Liu et al. 2009) and IL1RL1 (Nemeth et al. 2017). The up-regulation of these genes during inflammation is evident from the literature and supports our hypothesis that an inflammatory immune response at the ileum in the diarrhoea-susceptible sheep can increase severity of the disease.

On the other hand, it was surprising to observe up-regulation of several genes with anti-inflammatory roles: i) macrophage-derived chemokine (MDC/CCL22), a molecule thought to help prevent intestinal inflammation (Wang et al. 2009); ii) CCL22 and its receptor CCR4 (expressed by Th2-cells and Treg cells), known to play important roles in preventing inflammatory bowel diseases such as colitis (Akhabir and Sandford 2010) by promoting Treg cell communication with dendritic cells (Yuan et al. 2007; Rapp et al. 2019); iii) IL1RN binds to the IL-1 receptor but has a greater affinity than IL-1, so it blocks this major pro-inflammatory cytokine that is highly expressed in various acute and chronic inflammatory conditions (Dinarello 1998; Nicklin et al. 2000; Shiiba et al. 2015).

The polarity of the immune response is important with respect to the type of infection. As stated earlier, a Th2 immune response is desirable in helminth infection, but as the ileum is not the site of infection, it was surprising to observe the up-regulation of genes, including CD86, Ovar-DRB1, IL1RL1 and IL-6, that polarize the immune response towards a Th2 response. CD86 (also called B7-2) is expressed by antigen-presenting cells, such as dendritic cells and macrophages, and preferentially acts as a co-stimulator of the production of Th2 cells from Th precursor cells (Kuchroo et al. 1995). The up-regulation of CD86 in diarrhoea-susceptible sheep suggests that its role is to enhance IL-4 production, promoting a Th2 immune response (Zhou et al. 2015). Ovar-DRB1 codes for a major histocompatibility (MHC-II) molecule that is expressed by antigen-presenting cells (e.g. dendritic cells) that present antigenic molecules from helminths to naïve CD4^+^ T cells, so they can transform into Th2 cytokine-producing cells that lead the way to an antibody-producing, adaptive-immune response (McRae et al. 2015; Neefjes et al. 2011). Ovar-DRB1 paves the way towards this outcome, with up-regulation of IL-6 strengthening the response by blocking the Th1 response (Diehl et al. 2000). Another cytokine receptor, IL1RL1, is an important member of IL-1 receptor family and receptor for IL-33 (Zhou et al. 2015). The ‘IL-33 and its receptor IL1RL1 axis’ has been associated with inflammation and stimulation of the Th2 immune response in ulcerative colitis where levels of IL-33 are significantly increased (Nemeth et al. 2017; Akhabir and Sandford 2010). IL-6 has been shown to promote a Th2 response (Diehl et al. 2000; Diehl and Rincón 2002), a desirable outcome in helminth infection, but its pro-inflammatory properties could lead to chronic inflammation.

The functional enrichment analysis did not reveal links between any of the down-regulated genes and the promotion of the immune response or inflammation. Rather, most of the functions highlighted were related to muscle physiology and contraction, absorption and pathways preventing inflammation. The down-regulation of these biological processes and pathways suggests a disruption in, for example, peristalsis and the transmembrane ion transport system that are important for maintaining fluid balance in the GIT and are affected by inflammatory immune responses (Eisenhut 2006). Potassium ion (K^+^) transport is critical in fluid balance in the gut (Eisenhut 2006) and, in our study, is affected by down-regulation of ATP1A2. Enteric oxytocin plays significant roles in GIT physiology and the down-regulation of the oxytocin-signaling pathway in diarrhoea-susceptible sheep would contribute to increased inflammation, reduced transit time and increased faecal water content—in other words, the high diarrhoea outcome (Das et al. 2018; Welch et al. 2014). Cyclic adenosine monophosphate (cAMP) reduces gut inflammation by decreasing infiltration of leucocytes (Zimmerman et al. 2012), so the down-regulation of the cAMP-signaling pathway would also contribute to inflammation of the ileum, again promoting diarrhoea (Zimmerman et al. 2012; Schafer et al. 2010).

Some of the down-regulated genes in diarrhoea-susceptible sheep, including MYH11, CACNB2, ATP1A2, CAV3, PRKG1, FOXP2 and FBXO32, normally maintain a physiological and homeostatic environment in the gut, so their down-regulation would increase the severity of diarrhoea through, for example, disruption of gut absorptive function. MYH11 encodes myosin 11, a major contractile protein, that plays important roles in intracellular transport, signal transduction, cell migration and adhesion, and its down-regulation has been linked to poor prognosis in colorectal cancer (Wang et al. 2014). As most of the nutrients and fluids are absorbed from the epithelium of the small intestine, the various ion channels play critical roles (Das et al. 2018). For example, two genes, CACNB2 and CACNA1C, transcribe for voltage-dependent L-type calcium channels that are important for the influx of the calcium ions (Ca^+2^) required for intestinal smooth muscle contraction; selective blockage of these channels can lead to paralytic ileus (lack of movement in the intestine; (Das et al. 2018; Wegener et al. 2006). The genes ATP1A2 and ATP2B2 transcribe for ‘ATPase Na^+^/K^+^ transporting membrane polypeptides’ that maintain a concentration gradient for sodium (Na^+^) and potassium (K^+^) ions across the plasma membrane. These gradients are integral to physiological processes in many organ systems, including the gut, where they maintain electrolyte and fluid homeostasis (Das et al. 2018; Pirahanchi et al. 2020). CAV3 (caveolin-3) promotes vascular smooth muscle contraction and prevents atherosclerosis (Gutierrez-Pajares et al. 2015). PRKG1 transcribes for cGMP-dependent protein kinase (PKG-1) which promotes vasodilation and vascular permeability, but it is significantly down-regulated by inflammatory cytokines leading to vasoconstriction and a decreased vascular permeability, ultimately affecting nutrient absorption into the bloodstream (Browner et al. 2004). Overall, the functional enrichment of down-regulated genes in diarrhoea-susceptible sheep indicates a disruption of absorption and smooth muscle contraction and increased inflammation, all of which contribute to diarrhoea.

## Conclusion

In the diarrhoea-susceptible sheep, there were indicators of an inflammatory response through up-regulated genes enriched in biological processes and pathways, and the immune response was polarized towards a Th2 response. We conclude that this inflammatory response increases the severity of diarrhoea in susceptible sheep because genes with pro-inflammatory features dominate the genes with anti-inflammatory properties. On the other hand, all of the down-regulated genes and associated biological processes and pathways that were enriched were related to physiological processes like smooth muscle contraction, ion transport and the homeostasis of the gut environment. Nevertheless, it is clear that an enhanced inflammatory immune response, accompanied by down-regulation of ion transport, oxytocin and cAMP signaling, at sites other than where infection occurs, can contribute to the development of severe diarrhoea. Furthermore, there are clear similarities with inflammatory bowel disease in humans (e.g. Crohn’s disease) where an inflammatory immune response leads to Ileitis (inflammation in the ileum). While it is most evident that there is a hypersensitive immune response to helminth larvae in infected areas of GIT, more work is needed to determine whether an inflammatory response to intestinal microbiota, in the absence of helminths, also contributes to the expression of diarrhoea. With respect to managing the disease, we can target genes with anti-inflammatory roles can be to control the severity of diarrhoea.

## Supplementary Information

Supplementary Fig. S1 A heatmap illustrating the top 100 most significant differentially expressed genes in diarrhoea-susceptible and diarrhoea-resistant groups. (PNG 476 kb)

High resolution image (TIFF 5923 kb)

ESM 1 (XLSX 3594 kb)

ESM 2 (XLSX 107 kb)

## Data Availability

The raw sequencing reads generated in this study have been submitted to NCBI Gene Expression Omnibus (GEO) database under the BioProject accession number GSE179149.
